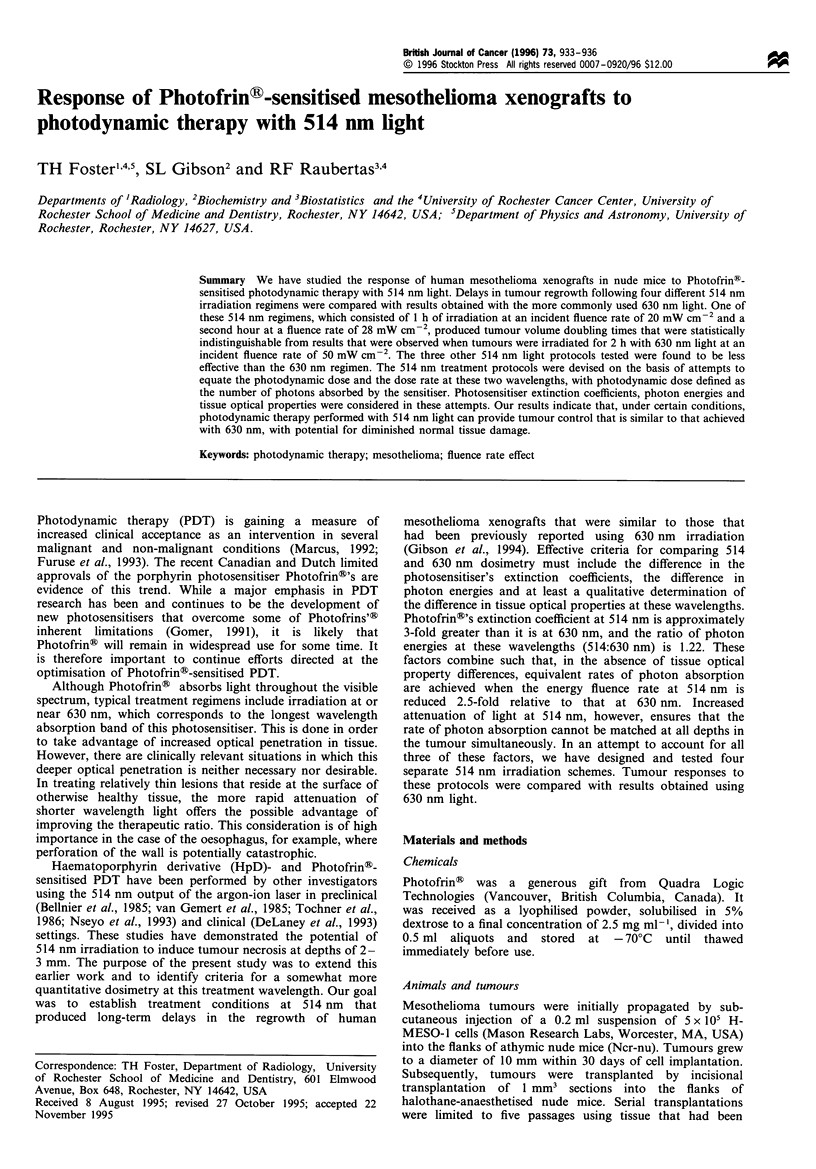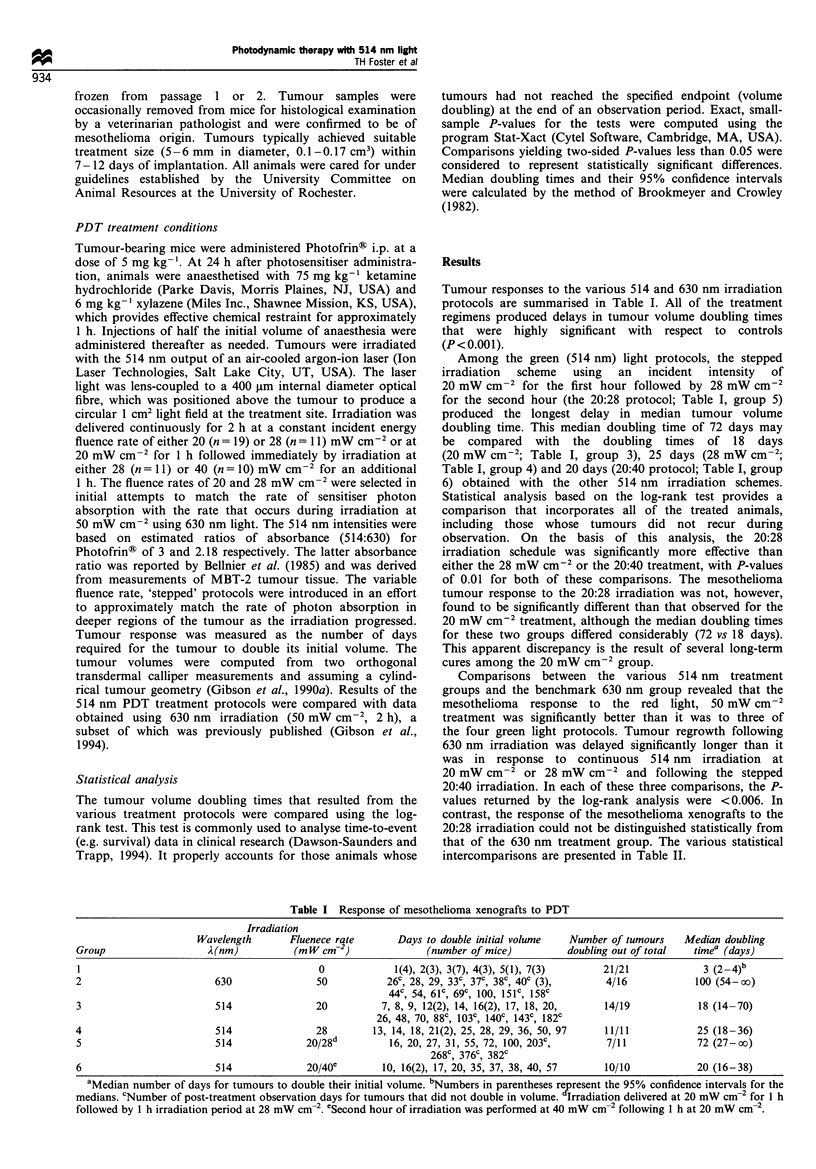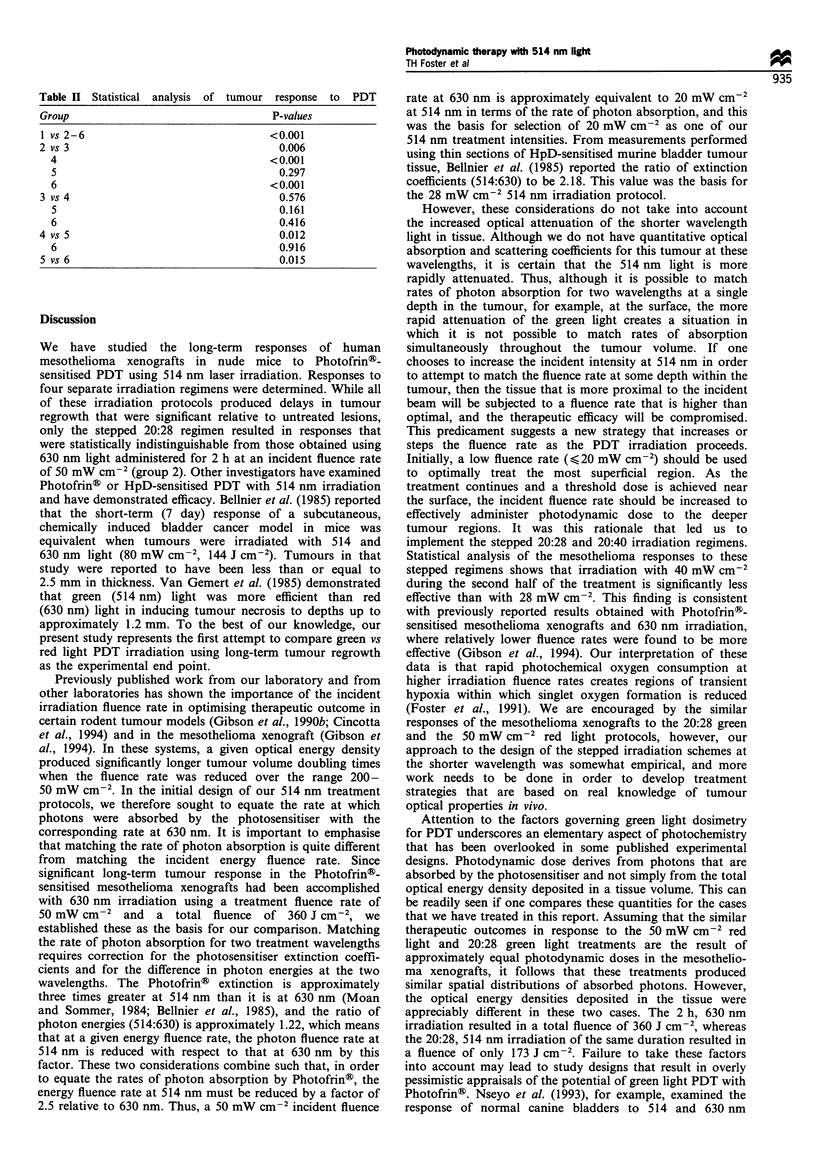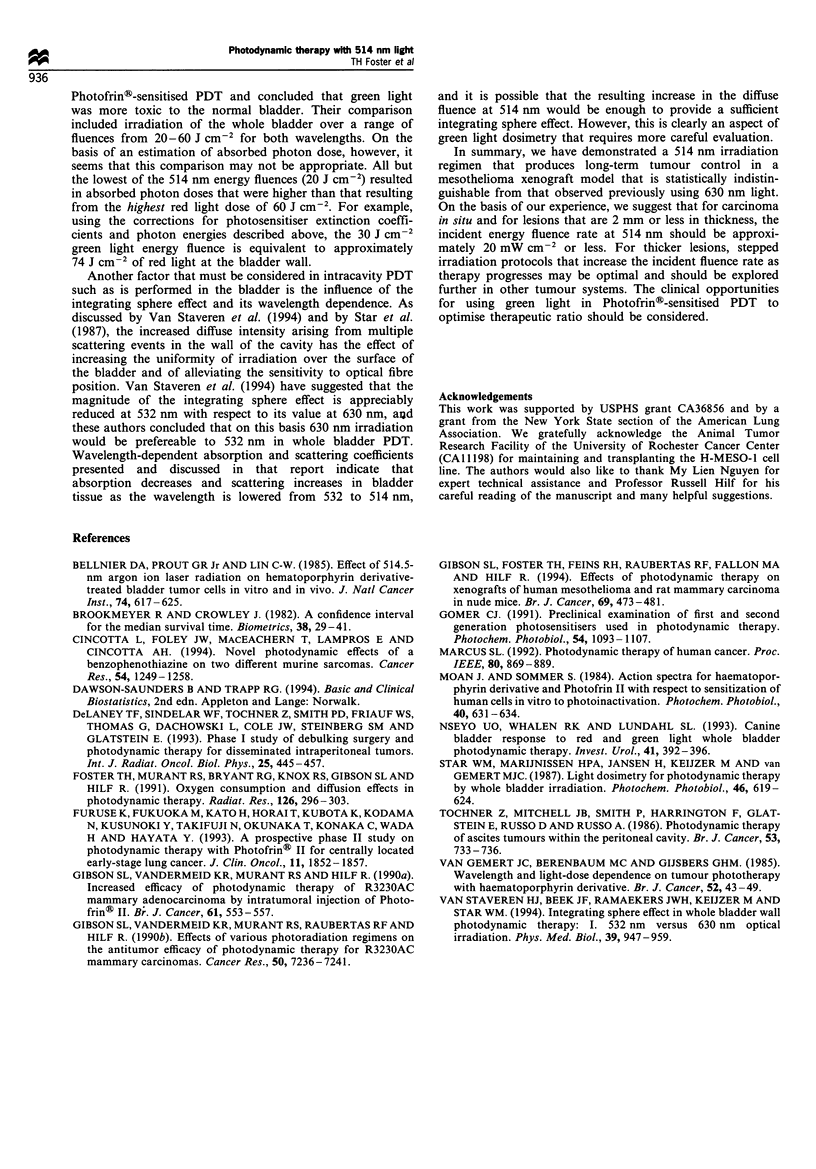# Response of Photofrin-sensitised mesothelioma xenografts to photodynamic therapy with 514 nm light.

**DOI:** 10.1038/bjc.1996.184

**Published:** 1996-04

**Authors:** T. H. Foster, S. L. Gibson, R. F. Raubertas

**Affiliations:** Department of Radiology, University of Rochester School of Medicine and Dentistry, NY 14642, USA.

## Abstract

We have studied the response of human mesothelioma xenografts in nude mice to Photofrin-sensitised photodynamic therapy with 514 nm light. Delays in tumour regrowth following four different 514 nm irradiation regimens were compared with results obtained with the more commonly used 630 nm light. One of these 514 nm regimens, which consisted of 1 h of irradiation at an incident fluence rate of 20 mW cm-2 and a second hour at a fluence rate of 28 mW cm-2, produced tumour volume doubling times that were statistically indistinguishable from results that were observed when tumours were irradiated for 2 h with 630 nm light at an incident fluence rate of 50 mW cm-2. The three other 514 nm light protocols tested were found to be less effective than the 630 nm regimen. The 514 nm treatment protocols were devised on the basis of attempts to equate the photodynamic dose and the dose rate at these two wavelengths, with photodynamic dose defined as the number of photons absorbed by the sensitiser. Photosensitiser extinction coefficients, photon energies and tissue optical properties were considered in these attempts. Our results indicate that, under certain conditions, photodynamic therapy performed with 514 nm light can provide tumour control that is similar to that achieved with 630 nm, with potential for diminished normal tissue damage.


British Journal of Cancer (1996) 73, 933-936
© 1996 Stockton Press All rights reserved 0007-0920/96 $12.00

### Response of Photofrin®-sensitised mesothelioma xenografts to photodynamic therapy with 514 nm light

TH Foster[1,4,5], SL Gibson[2] and RF Raubertas[3,4]

*Departments of [1]Radiology, [2]Biochemistry and [3]Biostatistics and the [4]University of Rochester Cancer Center, University of Rochester School of Medicine and Dentistry, Rochester, NY 14642, USA; [5]Department of Physics and Astronomy, University of Rochester, Rochester, NY 14627, USA.*

**Summary** We have studied the response of human mesothelioma xenografts in nude mice to Photofrin®-sensitised photodynamic therapy with 514 nm light. Delays in tumour regrowth following four different 514 nm irradiation regimens were compared with results obtained with the more commonly used 630 nm light. One of these 514 nm regimens, which consisted of 1 h of irradiation at an incident fluence rate of 20 mW $cm^{-2}$ and a second hour at a fluence rate of 28 mW $cm^{-2}$, produced tumour volume doubling times that were statistically indistinguishable from results that were observed when tumours were irradiated for 2 h with 630 nm light at an incident fluence rate of 50 mW $cm^{-2}$. The three other 514 nm light protocols tested were found to be less effective than the 630 nm regimen. The 514 nm treatment protocols were devised on the basis of attempts to equate the photodynamic dose and the dose rate at these two wavelengths, with photodynamic dose defined as the number of photons absorbed by the sensitiser. Photosensitiser extinction coefficients, photon energies and tissue optical properties were considered in these attempts. Our results indicate that, under certain conditions, photodynamic therapy performed with 514 nm light can provide tumour control that is similar to that achieved with 630 nm, with potential for diminished normal tissue damage.

**Keywords:** photodynamic therapy; mesothelioma; fluence rate effect

Photodynamic therapy (PDT) is gaining a measure of increased clinical acceptance as an intervention in several malignant and non-malignant conditions (Marcus, 1992; Furuse *et al.*, 1993). The recent Canadian and Dutch limited approvals of the porphyrin photosensitiser Photofrin®'s are evidence of this trend. While a major emphasis in PDT research has been and continues to be the development of new photosensitisers that overcome some of Photofrins'® inherent limitations (Gomer, 1991), it is likely that Photofrin® will remain in widespread use for some time. It is therefore important to continue efforts directed at the optimisation of Photofrin®-sensitised PDT.

Although Photofrin® absorbs light throughout the visible spectrum, typical treatment regimens include irradiation at or near 630 nm, which corresponds to the longest wavelength absorption band of this photosensitiser. This is done in order to take advantage of increased optical penetration in tissue. However, there are clinically relevant situations in which this deeper optical penetration is neither necessary nor desirable. In treating relatively thin lesions that reside at the surface of otherwise healthy tissue, the more rapid attenuation of shorter wavelength light offers the possible advantage of improving the therapeutic ratio. This consideration is of high importance in the case of the oesophagus, for example, where perforation of the wall is potentially catastrophic.

Haematoporphyrin derivative (HpD)- and Photofrin®-sensitised PDT have been performed by other investigators using the 514 nm output of the argon-ion laser in preclinical (Bellnier *et al.*, 1985; van Gemert *et al.*, 1985; Tochner *et al.*, 1986; Nseyo *et al.*, 1993) and clinical (DeLaney *et al.*, 1993) settings. These studies have demonstrated the potential of 514 nm irradiation to induce tumour necrosis at depths of 2–3 mm. The purpose of the present study was to extend this earlier work and to identify criteria for a somewhat more quantitative dosimetry at this treatment wavelength. Our goal was to establish treatment conditions at 514 nm that produced long-term delays in the regrowth of human mesothelioma xenografts that were similar to those that had been previously reported using 630 nm irradiation (Gibson *et al.*, 1994). Effective criteria for comparing 514 and 630 nm dosimetry must include the difference in the photosensitiser's extinction coefficients, the difference in photon energies and at least a qualitative determination of the difference in tissue optical properties at these wavelengths. Photofrin®'s extinction coefficient at 514 nm is approximately 3-fold greater than it is at 630 nm, and the ratio of photon energies at these wavelengths (514:630 nm) is 1.22. These factors combine such that, in the absence of tissue optical property differences, equivalent rates of photon absorption are achieved when the energy fluence rate at 514 nm is reduced 2.5-fold relative to that at 630 nm. Increased attenuation of light at 514 nm, however, ensures that the rate of photon absorption cannot be matched at all depths in the tumour simultaneously. In an attempt to account for all three of these factors, we have designed and tested four separate 514 nm irradiation schemes. Tumour responses to these protocols were compared with results obtained using 630 nm light.

### Materials and methods

### *Chemicals*

Photofrin® was a generous gift from Quadra Logic Technologies (Vancouver, British Columbia, Canada). It was received as a lyophilised powder, solubilised in 5% dextrose to a final concentration of 2.5 mg $ml^{-1}$, divided into 0.5 ml aliquots and stored at −70°C until thawed immediately before use.

### *Animals and tumours*

Mesothelioma tumours were initially propagated by subcutaneous injection of a 0.2 ml suspension of $5 \times 10^5$ H-MESO-1 cells (Mason Research Labs, Worcester, MA, USA) into the flanks of athymic nude mice (Ncr-nu). Tumours grew to a diameter of 10 mm within 30 days of cell implantation. Subsequently, tumours were transplanted by incisional transplantation of 1 $mm^3$ sections into the flanks of halothane-anaesthetised nude mice. Serial transplantations were limited to five passages using tissue that had been

Correspondence: TH Foster, Department of Radiology, University of Rochester School of Medicine and Dentistry, 601 Elmwood Avenue, Box 648, Rochester, NY 14642, USA
Received 8 August 1995; revised 27 October 1995; accepted 22 November 1995

frozen from passage 1 or 2. Tumour samples were occasionally removed from mice for histological examination by a veterinarian pathologist and were confirmed to be of mesothelioma origin. Tumours typically achieved suitable treatment size (5–6 mm in diameter, 0.1–0.17 $cm^3$) within 7–12 days of implantation. All animals were cared for under guidelines established by the University Committee on Animal Resources at the University of Rochester.

### *PDT treatment conditions*

Tumour-bearing mice were administered Photofrin® i.p. at a dose of 5 mg $kg^{-1}$. At 24 h after photosensitiser administration, animals were anaesthetised with 75 mg $kg^{-1}$ ketamine hydrochloride (Parke Davis, Morris Plaines, NJ, USA) and 6 mg $kg^{-1}$ xylazene (Miles Inc., Shawnee Mission, KS, USA), which provides effective chemical restraint for approximately 1 h. Injections of half the initial volume of anaesthesia were administered thereafter as needed. Tumours were irradiated with the 514 nm output of an air-cooled argon-ion laser (Ion Laser Technologies, Salt Lake City, UT, USA). The laser light was lens-coupled to a 400 μm internal diameter optical fibre, which was positioned above the tumour to produce a circular 1 $cm^2$ light field at the treatment site. Irradiation was delivered continuously for 2 h at a constant incident energy fluence rate of either 20 ($n$=19) or 28 ($n$=11) mW $cm^{-2}$ or at 20 mW $cm^{-2}$ for 1 h followed immediately by irradiation at either 28 ($n$=11) or 40 ($n$=10) mW $cm^{-2}$ for an additional 1 h. The fluence rates of 20 and 28 mW $cm^{-2}$ were selected in initial attempts to match the rate of sensitiser photon absorption with the rate that occurs during irradiation at 50 mW $cm^{-2}$ using 630 nm light. The 514 nm intensities were based on estimated ratios of absorbance (514:630) for Photofrin® of 3 and 2.18 respectively. The latter absorbance ratio was reported by Bellnier *et al.* (1985) and was derived from measurements of MBT-2 tumour tissue. The variable fluence rate, 'stepped' protocols were introduced in an effort to approximately match the rate of photon absorption in deeper regions of the tumour as the irradiation progressed. Tumour response was measured as the number of days required for the tumour to double its initial volume. The tumour volumes were computed from two orthogonal transdermal calliper measurements and assuming a cylindrical tumour geometry (Gibson *et al.*, 1990*a*). Results of the 514 nm PDT treatment protocols were compared with data obtained using 630 nm irradiation (50 mW $cm^{-2}$, 2 h), a subset of which was previously published (Gibson *et al.*, 1994).

### *Statistical analysis*

The tumour volume doubling times that resulted from the various treatment protocols were compared using the log-rank test. This test is commonly used to analyse time-to-event (e.g. survival) data in clinical research (Dawson-Saunders and Trapp, 1994). It properly accounts for those animals whose tumours had not reached the specified endpoint (volume doubling) at the end of an observation period. Exact, small-sample $P$-values for the tests were computed using the program Stat-Xact (Cytel Software, Cambridge, MA, USA). Comparisons yielding two-sided $P$-values less than 0.05 were considered to represent statistically significant differences. Median doubling times and their 95% confidence intervals were calculated by the method of Brookmeyer and Crowley (1982).

### Results

Tumour responses to the various 514 and 630 nm irradiation protocols are summarised in Table I. All of the treatment regimens produced delays in tumour volume doubling times that were highly significant with respect to controls ($P$<0.001).

Among the green (514 nm) light protocols, the stepped irradiation scheme using an incident intensity of 20 mW $cm^{-2}$ for the first hour followed by 28 mW $cm^{-2}$ for the second hour (the 20:28 protocol; Table I, group 5) produced the longest delay in median tumour volume doubling time. This median doubling time of 72 days may be compared with the doubling times of 18 days (20 mW $cm^{-2}$; Table I, group 3), 25 days (28 mW $cm^{-2}$; Table I, group 4) and 20 days (20:40 protocol; Table I, group 6) obtained with the other 514 nm irradiation schemes. Statistical analysis based on the log-rank test provides a comparison that incorporates all of the treated animals, including those whose tumours did not recur during observation. On the basis of this analysis, the 20:28 irradiation schedule was significantly more effective than either the 28 mW $cm^{-2}$ or the 20:40 treatment, with $P$-values of 0.01 for both of these comparisons. The mesothelioma tumour response to the 20:28 irradiation was not, however, found to be significantly different than that observed for the 20 mW $cm^{-2}$ treatment, although the median doubling times for these two groups differed considerably (72 *vs* 18 days). This apparent discrepancy is the result of several long-term cures among the 20 mW $cm^{-2}$ group.

Comparisons between the various 514 nm treatment groups and the benchmark 630 nm group revealed that the mesothelioma response to the red light, 50 mW $cm^{-2}$ treatment was significantly better than it was to three of the four green light protocols. Tumour regrowth following 630 nm irradiation was delayed significantly longer than it was in response to continuous 514 nm irradiation at 20 mW $cm^{-2}$ or 28 mW $cm^{-2}$ and following the stepped 20:40 irradiation. In each of these three comparisons, the $P$-values returned by the log-rank analysis were <0.006. In contrast, the response of the mesothelioma xenografts to the 20:28 irradiation could not be distinguished statistically from that of the 630 nm treatment group. The various statistical intercomparisons are presented in Table II.

**Table I** Response of mesothelioma xenografts to PDT

| Group | *Irradiation* Wavelength λ(nm) | Fluenece rate (mW $cm^{-2}$) | Days to double initial volume (number of mice) | Number of tumours doubling out of total | Median doubling time[a] (days) |
|---|---|---|---|---|---|
| 1 | | 0 | 1(4), 2(3), 3(7), 4(3), 5(1), 7(3) | 21/21 | 3 (2–4)[b] |
| 2 | 630 | 50 | 26[c], 28, 29, 33[c], 37[c], 38[c], 40[c] (3), 44[c], 54, 61[c], 69[c], 100, 151[c], 158[c] | 4/16 | 100 (54–∞) |
| 3 | 514 | 20 | 7, 8, 9, 12(2), 14, 16(2), 17, 18, 20, 26, 48, 70, 88[c], 103[c], 140[c], 143[c], 182[c] | 14/19 | 18 (14–70) |
| 4 | 514 | 28 | 13, 14, 18, 21(2), 25, 28, 29, 36, 50, 97 | 11/11 | 25 (18–36) |
| 5 | 514 | 20/28[d] | 16, 20, 27, 31, 55, 72, 100, 203[c], 268[c], 376[c], 382[c] | 7/11 | 72 (27–∞) |
| 6 | 514 | 20/40[e] | 10, 16(2), 17, 20, 35, 37, 38, 40, 57 | 10/10 | 20 (16–38) |

[a]Median number of days for tumours to double their initial volume. [b]Numbers in parentheses represent the 95% confidence intervals for the medians. [c]Number of post-treatment observation days for tumours that did not double in volume. [d]Irradiation delivered at 20 mW $cm^{-2}$ for 1 h followed by 1 h irradiation period at 28 mW $cm^{-2}$. [e]Second hour of irradiation was performed at 40 mW $cm^{-2}$ following 1 h at 20 mW $cm^{-2}$.

**Table II** Statistical analysis of tumour response to PDT

| Group | P-values |
|---|---|
| 1 *vs* 2–6 | <0.001 |
| 2 *vs* 3 | 0.006 |
| 4 | <0.001 |
| 5 | 0.297 |
| 6 | <0.001 |
| 3 *vs* 4 | 0.576 |
| 5 | 0.161 |
| 6 | 0.416 |
| 4 *vs* 5 | 0.012 |
| 6 | 0.916 |
| 5 *vs* 6 | 0.015 |

### Discussion

We have studied the long-term responses of human mesothelioma xenografts in nude mice to Photofrin®-sensitised PDT using 514 nm laser irradiation. Responses to four separate irradiation regimens were determined. While all of these irradiation protocols produced delays in tumour regrowth that were significant relative to untreated lesions, only the stepped 20:28 regimen resulted in responses that were statistically indistinguishable from those obtained using 630 nm light administered for 2 h at an incident fluence rate of 50 mW cm$^{-2}$ (group 2). Other investigators have examined Photofrin® or HpD-sensitised PDT with 514 nm irradiation and have demonstrated efficacy. Bellnier *et al.* (1985) reported that the short-term (7 day) response of a subcutaneous, chemically induced bladder cancer model in mice was equivalent when tumours were irradiated with 514 and 630 nm light (80 mW cm$^{-2}$, 144 J cm$^{-2}$). Tumours in that study were reported to have been less than or equal to 2.5 mm in thickness. Van Gemert *et al.* (1985) demonstrated that green (514 nm) light was more efficient than red (630 nm) light in inducing tumour necrosis to depths up to approximately 1.2 mm. To the best of our knowledge, our present study represents the first attempt to compare green *vs* red light PDT irradiation using long-term tumour regrowth as the experimental end point.

Previously published work from our laboratory and from other laboratories has shown the importance of the incident irradiation fluence rate in optimising therapeutic outcome in certain rodent tumour models (Gibson *et al.*, 1990*b*; Cincotta *et al.*, 1994) and in the mesothelioma xenograft (Gibson *et al.*, 1994). In these systems, a given optical energy density produced significantly longer tumour volume doubling times when the fluence rate was reduced over the range 200–50 mW cm$^{-2}$. In the initial design of our 514 nm treatment protocols, we therefore sought to equate the rate at which photons were absorbed by the photosensitiser with the corresponding rate at 630 nm. It is important to emphasise that matching the rate of photon absorption is quite different from matching the incident energy fluence rate. Since significant long-term tumour response in the Photofrin®-sensitised mesothelioma xenografts had been accomplished with 630 nm irradiation using a treatment fluence rate of 50 mW cm$^{-2}$ and a total fluence of 360 J cm$^{-2}$, we established these as the basis for our comparison. Matching the rate of photon absorption for two treatment wavelengths requires correction for the photosensitiser extinction coefficients and for the difference in photon energies at the two wavelengths. The Photofrin® extinction is approximately three times greater at 514 nm than it is at 630 nm (Moan and Sommer, 1984; Bellnier *et al.*, 1985), and the ratio of photon energies (514:630) is approximately 1.22, which means that at a given energy fluence rate, the photon fluence rate at 514 nm is reduced with respect to that at 630 nm by this factor. These two considerations combine such that, in order to equate the rates of photon absorption by Photofrin®, the energy fluence rate at 514 nm must be reduced by a factor of 2.5 relative to 630 nm. Thus, a 50 mW cm$^{-2}$ incident fluence rate at 630 nm is approximately equivalent to 20 mW cm$^{-2}$ at 514 nm in terms of the rate of photon absorption, and this was the basis for selection of 20 mW cm$^{-2}$ as one of our 514 nm treatment intensities. From measurements performed using thin sections of HpD-sensitised murine bladder tumour tissue, Bellnier *et al.* (1985) reported the ratio of extinction coefficients (514:630) to be 2.18. This value was the basis for the 28 mW cm$^{-2}$ 514 nm irradiation protocol.

However, these considerations do not take into account the increased optical attenuation of the shorter wavelength light in tissue. Although we do not have quantitative optical absorption and scattering coefficients for this tumour at these wavelengths, it is certain that the 514 nm light is more rapidly attenuated. Thus, although it is possible to match rates of photon absorption for two wavelengths at a single depth in the tumour, for example, at the surface, the more rapid attenuation of the green light creates a situation in which it is not possible to match rates of absorption simultaneously throughout the tumour volume. If one chooses to increase the incident intensity at 514 nm in order to attempt to match the fluence rate at some depth within the tumour, then the tissue that is more proximal to the incident beam will be subjected to a fluence rate that is higher than optimal, and the therapeutic efficacy will be compromised. This predicament suggests a new strategy that increases or steps the fluence rate as the PDT irradiation proceeds. Initially, a low fluence rate ($\leqslant$20 mW cm$^{-2}$) should be used to optimally treat the most superficial region. As the treatment continues and a threshold dose is achieved near the surface, the incident fluence rate should be increased to effectively administer photodynamic dose to the deeper tumour regions. It was this rationale that led us to implement the stepped 20:28 and 20:40 irradiation regimens. Statistical analysis of the mesothelioma responses to these stepped regimens shows that irradiation with 40 mW cm$^{-2}$ during the second half of the treatment is significantly less effective than with 28 mW cm$^{-2}$. This finding is consistent with previously reported results obtained with Photofrin®-sensitised mesothelioma xenografts and 630 nm irradiation, where relatively lower fluence rates were found to be more effective (Gibson *et al.*, 1994). Our interpretation of these data is that rapid photochemical oxygen consumption at higher irradiation fluence rates creates regions of transient hypoxia within which singlet oxygen formation is reduced (Foster *et al.*, 1991). We are encouraged by the similar responses of the mesothelioma xenografts to the 20:28 green and the 50 mW cm$^{-2}$ red light protocols, however, our approach to the design of the stepped irradiation schemes at the shorter wavelength was somewhat empirical, and more work needs to be done in order to develop treatment strategies that are based on real knowledge of tumour optical properties *in vivo*.

Attention to the factors governing green light dosimetry for PDT underscores an elementary aspect of photochemistry that has been overlooked in some published experimental designs. Photodynamic dose derives from photons that are absorbed by the photosensitiser and not simply from the total optical energy density deposited in a tissue volume. This can be readily seen if one compares these quantities for the cases that we have treated in this report. Assuming that the similar therapeutic outcomes in response to the 50 mW cm$^{-2}$ red light and 20:28 green light treatments are the result of approximately equal photodynamic doses in the mesothelioma xenografts, it follows that these treatments produced similar spatial distributions of absorbed photons. However, the optical energy densities deposited in the tissue were appreciably different in these two cases. The 2 h, 630 nm irradiation resulted in a total fluence of 360 J cm$^{-2}$, whereas the 20:28, 514 nm irradiation of the same duration resulted in a fluence of only 173 J cm$^{-2}$. Failure to take these factors into account may lead to study designs that result in overly pessimistic appraisals of the potential of green light PDT with Photofrin®. Nseyo *et al.* (1993), for example, examined the response of normal canine bladders to 514 and 630 nm

Photofrin®-sensitised PDT and concluded that green light was more toxic to the normal bladder. Their comparison included irradiation of the whole bladder over a range of fluences from 20–60 J $cm^{-2}$ for both wavelengths. On the basis of an estimation of absorbed photon dose, however, it seems that this comparison may not be appropriate. All but the lowest of the 514 nm energy fluences (20 J $cm^{-2}$) resulted in absorbed photon doses that were higher than that resulting from the *highest* red light dose of 60 J $cm^{-2}$. For example, using the corrections for photosensitiser extinction coefficients and photon energies described above, the 30 J $cm^{-2}$ green light energy fluence is equivalent to approximately 74 J $cm^{-2}$ of red light at the bladder wall.

Another factor that must be considered in intracavity PDT such as is performed in the bladder is the influence of the integrating sphere effect and its wavelength dependence. As discussed by Van Staveren *et al.* (1994) and by Star *et al.* (1987), the increased diffuse intensity arising from multiple scattering events in the wall of the cavity has the effect of increasing the uniformity of irradiation over the surface of the bladder and of alleviating the sensitivity to optical fibre position. Van Staveren *et al.* (1994) have suggested that the magnitude of the integrating sphere effect is appreciably reduced at 532 nm with respect to its value at 630 nm, and these authors concluded that on this basis 630 nm irradiation would be prefereable to 532 nm in whole bladder PDT. Wavelength-dependent absorption and scattering coefficients presented and discussed in that report indicate that absorption decreases and scattering increases in bladder tissue as the wavelength is lowered from 532 to 514 nm, and it is possible that the resulting increase in the diffuse fluence at 514 nm would be enough to provide a sufficient integrating sphere effect. However, this is clearly an aspect of green light dosimetry that requires more careful evaluation.

In summary, we have demonstrated a 514 nm irradiation regimen that produces long-term tumour control in a mesothelioma xenograft model that is statistically indistinguishable from that observed previously using 630 nm light. On the basis of our experience, we suggest that for carcinoma *in situ* and for lesions that are 2 mm or less in thickness, the incident energy fluence rate at 514 nm should be approximately 20 mW $cm^{-2}$ or less. For thicker lesions, stepped irradiation protocols that increase the incident fluence rate as therapy progresses may be optimal and should be explored further in other tumour systems. The clinical opportunities for using green light in Photofrin®-sensitised PDT to optimise therapeutic ratio should be considered.

**Acknowledgements**
This work was supported by USPHS grant CA36856 and by a grant from the New York State section of the American Lung Association. We gratefully acknowledge the Animal Tumor Research Facility of the University of Rochester Cancer Center (CA11198) for maintaining and transplanting the H-MESO-1 cell line. The authors would also like to thank My Lien Nguyen for expert technical assistance and Professor Russell Hilf for his careful reading of the manuscript and many helpful suggestions.

### References

BELLNIER DA, PROUT GR Jr AND LIN C-W. (1985). Effect of 514.5-nm argon ion laser radiation on hematoporphyrin derivative-treated bladder tumor cells in vitro and in vivo. *J. Natl Cancer Inst.*, **74**, 617–625.

BROOKMEYER R AND CROWLEY J. (1982). A confidence interval for the median survival time. *Biometrics*, **38**, 29–41.

CINCOTTA L, FOLEY JW, MACEACHERN T, LAMPROS E AND CINCOTTA AH. (1994). Novel photodynamic effects of a benzophenothiazine on two different murine sarcomas. *Cancer Res.*, **54**, 1249–1258.

DAWSON-SAUNDERS B AND TRAPP RG. (1994). *Basic and Clinical Biostatistics*, 2nd edn. Appleton and Lange: Norwalk.

DeLANEY TF, SINDELAR WF, TOCHNER Z, SMITH PD, FRIAUF WS, THOMAS G, DACHOWSKI L, COLE JW, STEINBERG SM AND GLATSTEIN E. (1993). Phase I study of debulking surgery and photodynamic therapy for disseminated intraperitoneal tumors. *Int. J. Radiat. Oncol. Biol. Phys.*, **25**, 445–457.

FOSTER TH, MURANT RS, BRYANT RG, KNOX RS, GIBSON SL AND HILF R. (1991). Oxygen consumption and diffusion effects in photodynamic therapy. *Radiat. Res.*, **126**, 296–303.

FURUSE K, FUKUOKA M, KATO H, HORAI T, KUBOTA K, KODAMA N, KUSUNOKI Y, TAKIFUJI N, OKUNAKA T, KONAKA C, WADA H AND HAYATA Y. (1993). A prospective phase II study on photodynamic therapy with Photofrin® II for centrally located early-stage lung cancer. *J. Clin. Oncol.*, **11**, 1852–1857.

GIBSON SL, VANDERMEID KR, MURANT RS AND HILF R. (1990*a*). Increased efficacy of photodynamic therapy of R3230AC mammary adenocarcinoma by intratumoral injection of Photofrin® II. *Br. J. Cancer*, **61**, 553–557.

GIBSON SL, VANDERMEID KR, MURANT RS, RAUBERTAS RF AND HILF R. (1990*b*). Effects of various photoradiation regimens on the antitumor efficacy of photodynamic therapy for R3230AC mammary carcinomas. *Cancer Res.*, **50**, 7236–7241.

GIBSON SL, FOSTER TH, FEINS RH, RAUBERTAS RF, FALLON MA AND HILF R. (1994). Effects of photodynamic therapy on xenografts of human mesothelioma and rat mammary carcinoma in nude mice. *Br. J. Cancer*, **69**, 473–481.

GOMER CJ. (1991). Preclinical examination of first and second generation photosensitisers used in photodynamic therapy. *Photochem. Photobiol.*, **54**, 1093–1107.

MARCUS SL. (1992). Photodynamic therapy of human cancer. *Proc. IEEE*, **80**, 869–889.

MOAN J. AND SOMMER S. (1984). Action spectra for haematoporphyrin derivative and Photofrin II with respect to sensitization of human cells in vitro to photoinactivation. *Photochem. Photobiol.*, **40**, 631–634.

NSEYO UO, WHALEN RK AND LUNDAHL SL. (1993). Canine bladder response to red and green light whole bladder photodynamic therapy. *Invest. Urol.*, **41**, 392–396.

STAR WM, MARIJNISSEN HPA, JANSEN H, KEIJZER M AND van GEMERT MJC. (1987). Light dosimetry for photodynamic therapy by whole bladder irradiation. *Photochem. Photobiol.*, **46**, 619–624.

TOCHNER Z, MITCHELL JB, SMITH P, HARRINGTON F, GLATSTEIN E, RUSSO D AND RUSSO A. (1986). Photodynamic therapy of ascites tumours within the peritoneal cavity. *Br. J. Cancer*, **53**, 733–736.

VAN GEMERT JC, BERENBAUM MC AND GIJSBERS GHM. (1985). Wavelength and light-dose dependence on tumour phototherapy with haematoporphyrin derivative. *Br. J. Cancer*, **52**, 43–49.

VAN STAVEREN HJ, BEEK JF, RAMAEKERS JWH, KEIJZER M AND STAR WM. (1994). Integrating sphere effect in whole bladder wall photodynamic therapy: I. 532 nm versus 630 nm optical irradiation. *Phys. Med. Biol.*, **39**, 947–959.